# Supplementary material for: Centre of pressure changes during stance but not during gait in young women after alcohol intoxication
Source: PeerJ. 2023 Nov 29;11:e16511. doi: 10.7717/peerj.16511 (PMC10693231; doi:10.7717/peerj.16511)
Supplement: Supplemental Information 1 [file peerj-11-16511-s001.pdf]

Participant (code)	Stance					
	Gender	setting: sober=s, intoxicated=i	Analysis time, sec	COP path length, mm	COP average velocity, mm/sec	Deviation X, mm
ALKOHOL 003	female	i	30	354,8	11,827	18,491
ALKOHOL 007	female	i	30	718,021	23,934	21,515
ALKOHOL 008	female	i	30	360,646	12,022	23,182
ALKOHOL 018	female	i	30	1153,461	38,449	0,45
ALKOHOL 026	female	i	30	569,229	18,974	10,205
ALKOHOL 030	female	i	30	536,231	17,874	35,727
ALKOHOL036	female	i	30	891,341	29,711	25,166
ALKOHOL037	female	i	30	612,243	20,408	6,59
ALKOHOL041	female	i	30	444,914	14,83	26,297
ALKOHOL048	female	i	30	435,707	14,524	13,665
ALKOHOL050	female	i	30	302,076	10,069	23,806
ALKOHOL052	female	i	30	562,191	18,74	25,899
ALKOHOL053	female	i	30	532,694	17,756	25,172
ALKOHOL062	female	i	30	776,344	25,878	26,356
ALKOHOL065	female	i	30	500,475	16,682	11,279
ALKOHOL066	female	i	30	517,116	17,237	22,772
ALKOHOL067	female	i	30	334,583	11,153	13,577
ALKOHOL080	female	i	30	636,881	21,229	28,171
ALKOHOL081	female	i	30	288,704	9,623	28,913
ALKOHOL091	female	i	30	461,815	15,394	10,1
ALKOHOL092	female	i	30	471,765	15,725	26,315
ALKOHOL098	female	i	30	481,422	16,047	11,916
ALKOHOL099	female	i	30	522,65	17,422	15,524
ALKOHOL102	female	i	30	352,288	11,743	20,902
ALKOHOL103	female	i	30	346,772	11,559	38,518
ALKOHOL104	female	i	30	256,389	8,546	31,399
Alkohol110	female	i	30	387,006	12,9	-1,253
Alkohol112	female	i	30	686,934	22,898	7,784
AKLKOHOL100	female	i	30	342,942	11,431	16,867
ALKOHOL039	female	i	30	522,8	17,427	32,796
ALKOHOL081	female	s	30	163,498	5,45	37,459
ALKOHOL 003	female	s	30	338,287	11,276	24,761
ALKOHOL 007	female	s	30	326,403	10,88	24,461
ALKOHOL 008	female	s	30	319,099	10,637	11,843
ALKOHOL 018	female	s	30	433,235	14,441	9,409
ALKOHOL 026	female	s	30	513,281	17,109	2,329
ALKOHOL 030	female	s	30	276,956	9,232	21,387
ALKOHOL036	female	s	30	256,299	8,543	17,577
ALKOHOL037	female	s	30	300,057	10,002	17,925
ALKOHOL039	female	s	30	287,097	9,57	37,107
ALKOHOL041	female	s	30	293,231	9,774	18,505
ALKOHOL048	female	s	30	268,827	8,961	13,605
ALKOHOL050	female	s	30	235,601	7,853	12,592

ALKOHOL052	female	s	30	213,556	7,119	22,333
ALKOHOL053	female	s	30	302,616	10,087	20,515
ALKOHOL062	female	s	30	239,125	7,971	28,111
ALKOHOL065	female	s	30	350,785	11,693	3,641
ALKOHOL066	female	s	30	266,415	8,881	14,492
ALKOHOL067	female	s	30	268,325	8,944	19,511
ALKOHOL080	female	s	30	223,672	7,456	27,112
ALKOHOL091	female	s	30	275,153	9,172	11,711
ALKOHOL092	female	s	30	275,529	9,184	16,617
ALKOHOL098	female	s	30	326,303	10,877	11,574
ALKOHOL099	female	s	30	425,66	14,189	20,923
ALKOHOL102	female	s	30	276,521	9,217	34,689
ALKOHOL103	female	s	30	233,832	7,794	32,866
ALKOHOL104	female	s	30	190,025	6,334	19,476
Alkohol110	female	s	30	215,834	7,194	14,013
Alkohol112	female	s	30	339,406	11,314	17,036
AKLKOHOL100	female	s	30	229,369	7,646	13,199

Deviation Y, mm	Age (years)	height (cm)	body mass (kg)	BrAC (promile)	AUDIT score	setting: sober=s, intoxicated=i	Stride length, cm	Step width, cm
-6,197	23,83	163,9	56	1,13	5	i	128,7	4,791
-10,328	25,33	169,4	72,1	1,16	10	i	156,2	11,39
-7,845	28,62	172,1	63,7	1,13	9	i	141,4	8,713
-7,034	24,58	170,5	61	1,19	7	i	148,2	8,704
-1,501	23,13	157	63,5	1,15	7	i	127	8,49
-11,705	30,27	168	59,5	1,12	6	i	123,6	9,998
-4,675	23,83	167,4	72,1	1,01	9	i	127,9	10,69
-3,865	20,72	158,7	55,9	1,05	5	i	127	11,67
-20,303	24,12	170	64,7	1,07	4	i	116	6,413
-6,051	23,63	176,1	78,1	1,14	5	i	149,9	9,789
-10,348	22,29	170,4	59,7	1,08	3	i	132,1	10,57
2,071	23,00	166,5	63,5	1,12	5	i	131,3	11,58
-4,323	20,56	170,7	69,5	1,17	10	i	109,7	11,93
-8,564	23,34	176,5	71,4	1,1	4	i	107,1	10,65
-4,377	28,97	174	63,9	0,99	10	i	128,7	11,49
-7,495	30,35	169,1	65,8	1,04	6	i	128,7	10,59
-9,461	20,33	161,9	63,2	1,08	13	i	149,1	5,998
-8,089	26,50	168	66,3	1,17	5	i	125,3	12,33
-7,179	27,21	161	64,1	1,16	9	i	106,3	6,503
-5,638	20,91	161	55	0,99	9	i	139,3	11,81
-2,077	20,36	172,3	66,8	1,05	11	i	127,9	3,292
4,292	21,30	171,6	58,6	1,11	10	i	131,3	6,426
-14,115	20,33	168,7	64,1	1,08	4	i	121,5	15,3
0,549	23,87	161	61,9	0,99	6	i	151,6	10,73
-9,117	22,12	177,2	69,8	1,02	8	i	134,7	7,769
-3,556	22,34	170,9	68,6	1,12	10	i	146,5	9,504
5,246	26,44	162,6	61,8	0,99	5	i	145,7	10,15
-3,726	22,79	177,3	79,5	1,17	5	i	164,3	10,46
2,898	26,47	162,5	73,9	1,1	7	i	134,7	10,33
-9,705	24,43	165,4	53,5	1,15	3	i	146,5	6,887
-9,896	27,21	161	64,1	1,16	9	s	107	5,113
-1,655	23,83	163,9	56	1,13	5	s	122,8	6,962
1,285	25,33	169,4	72,1	1,16	10	s	139,7	9,9
1,575	28,62	172,1	63,7	1,13	9	s	135,9	8,509
-8,509	24,58	170,5	61	1,19	7	s	145,7	8,586
-3,375	23,13	157	63,5	1,15	7	s	115,2	10,22
-0,646	30,27	168	59,5	1,12	6	s	133,8	9,302
-9,258	23,83	167,4	72,1	1,01	9	s	127,9	11,38
-4,865	20,72	158,7	55,9	1,05	5	s	115,7	11,29
-10,253	24,43	165,4	53,5	1,15	3	s	133,8	9,417
-17,554	24,12	170	64,7	1,07	4	s	113,1	4,525
-9,739	23,63	176,1	78,1	1,14	5	s	138,9	12,07
-1,327	22,29	170,4	59,7	1,08	3	s	131,3	10,27

-5,349	23,00	166,5	63,5	1,12	5	s	128,7	10,12
-6,249	20,56	170,7	69,5	1,17	10	s	107,1	10,29
-14,836	23,34	176,5	71,4	1,1	4	s	103,3	10,78
0,255	28,97	174	63,9	0,99	10	s	138,9	10,62
-14,533	30,35	169,1	65,8	1,04	6	s	120,3	8,248
-7,555	20,33	161,9	63,2	1,08	13	s	144	6,954
-2,852	26,50	168	66,3	1,17	5	s	113,5	10,66
-12,135	20,91	161	55	0,99	9	s	132,1	11,31
0,316	20,36	172,3	66,8	1,05	11	s	127	6,867
6,879	21,30	171,6	58,6	1,11	10	s	129,6	8,898
-11,378	20,33	168,7	64,1	1,08	4	s	115,2	13,69
-0,154	23,87	161	61,9	0,99	6	s	132,1	8,775
-10,959	22,12	177,2	69,8	1,02	8	s	127,5	8,471
0,589	22,34	170,9	68,6	1,12	10	s	135,5	9,558
-12,089	26,44	162,6	61,8	0,99	5	s	144,8	10,77
-0,953	22,79	177,3	79,5	1,17	5	s	150,7	7,547
-5,649	26,47	162,5	73,9	1,1	7	s	121,1	6,732

Gait

Cadence, steps/min	Velocity, km/h	Length of gait line L, mm	Length of gait line R, mm	Single limb support line L, mm	Single limb support line R, mm	Ant/post position, mm	Ant/post position SD, mm
110,438	4,237	210,534	211,004	124,914	97,546	-11,534	1,686
100,395	4,705	239,724	229,378	126,835	123,834	-2,989	7,075
110,559	4,69	218,395	211,575	118,211	124,053	3,578	5,76
109,091	4,915	212,247	221,955	126,791	147,841	-7,058	3,526
108,466	4,077	219,172	209,398	120,037	126,411	-3,768	1,508
108,442	4,049	221,84	227,861	109,912	122,613	4,338	2,272
104,658	3,935	226,883	223,832	96,27	108,087	3,711	2,342
104,658	3,988	208,641	216,429	105,589	114,202	4,797	0,688
84,552	3,003	234,532	232,194	129,209	114,277	5,698	4,928
98,77	4,434	232,534	249,657	122,114	77,154	15,317	10,292
107,143	4,256	215,023	216,507	134,207	122,106	5,6	1,573
98,907	3,878	217,195	208,824	129,683	122,336	3,805	2,307
84,593	2,778	221,642	223,688	101,876	114,285	12,406	4,493
89,838	2,891	232,702	217,675	120,55	57,572	-18,442	18,581
90,415	3,578	256,072	245,949	161,853	145,045	-6,442	3,03
107,143	4,147	171,297	219,401	108,19	122,575	-5,31	1,513
111,111	4,959	218,155	229,457	107,742	115,942	5,523	0,795
106,042	4,033	165,849	151,717	93,285	88,047	12,632	25,764
106,425	3,383	203,278	214,709	106,549	105,378	-0,131	4,158
115,531	4,874	203,98	199,107	124,154	108,299	6,618	0,754
101,695	3,867	174,642	223,377	82,969	126,27	-3,083	4,652
112,509	4,459	222,326	216,723	125,447	124,805	-9,698	1,513
123,475	4,568	213,634	189,369	133,928	130,464	-0,818	1,668
120,036	5,452	222,674	222,997	137,767	130,538	-0,624	5,378
110,438	4,452	228,671	228,54	108,944	116,2	4,66	0,813
101,714	4,41	190,611	235,769	132,501	131,459	1,612	4,766
119,248	5,201	220,782	164,826	105,022	99,446	30,544	19,221
133,333	6,551	223,238	220,802	128,787	118,253	-1,924	3,964
107,166	4,22	225,531	227,82	130,352	124,056	8,673	2,778
119,248	5,231	219,788	223,841	124,532	136,496	6,709	2,286
90,15	2,895	214,866	217,244	109,644	112,489	0,842	3,498
110,559	4,064	207,659	208,988	99,074	114,316	-7,84	2,79
93,21	3,755	231,749	228,858	115,133	120,539	5,416	3,649
111,635	4,574	218,202	210,026	111,784	117,13	4,194	0,831
118,462	5,256	205,207	224,893	123,981	125,731	-1,329	0,853
113,287	4,085	215,662	153,708	127,619	45,046	15,044	9,178
110,438	4,443	219,778	226,154	111,288	121,325	-1,054	2,752
103,448	3,977	225,232	222,19	114,413	110,069	10,736	0,966
101,159	3,504	211,956	211,73	107,174	110,778	2,316	4,758
121,609	4,892	214,598	220,977	121,597	137,986	11,481	0,875
91,269	3,088	241,273	227,115	135,913	111,86	0,703	4,161
98,361	4,09	227,226	231,479	102,366	132,811	21,65	2,64
111,81	4,432	214,341	211,416	129,791	113,653	15,613	2,756

95,783	3,683	210,82	214,948	111,737	128,336	1,162	2,04
88,842	2,872	214,117	222,699	110,851	118,195	6,921	2,375
88,913	2,745	225,229	219,49	130,922	54,038	-10,949	11,612
96,774	4,032	257,47	255,608	176,537	150,432	-2,646	0,409
101,695	3,738	172,449	218,853	93,529	129,565	4,29	1,584
109,764	4,713	219,704	226,802	116,229	131,523	0,245	0,719
100,35	3,437	114,926	140,579	97,499	102,208	42,169	8,479
113,933	4,476	203,293	200,177	96,815	111,588	5,591	2,389
102,808	3,909	207,514	224,482	121,407	112,325	-5,583	3,851
106,285	4,049	217,866	214,143	111,692	112,124	-1,695	3,33
122,483	4,187	212,046	205,849	130,706	130,368	-3,738	3,548
117,647	4,663	221,82	223,43	136,079	131,764	0,609	2,243
110,333	4,183	228,716	226,511	106,971	115,333	8,45	2,724
104,658	4,255	229,945	229,577	128,955	126,423	7,05	0,793
125	5,431	153,869	212,663	96,179	121,145	9,354	38,351
120,12	5,512	230,218	209,375	99,639	113,216	5,941	1,983
95,622	3,523	226,44	228,47	121,793	103,964	6,983	0,316

Lateral symmetry, mm	Lateral symmetry SD, mm
7,507	2,771
5,253	5,195
-0,182	4,781
-3,815	3,279
-3,619	0,746
-1,927	1,714
0,338	6,264
-7,411	0,454
2,054	3,554
2,273	6,536
-3,714	1,479
1,831	2,685
-0,796	2,823
23,308	18,618
3,404	5,42
-4,852	3,46
-5,047	0,445
-1,016	29,389
-1,696	5,028
2,553	1,857
-3,447	2,797
-3,953	1,088
-0,545	1,212
1,279	4,481
-6,053	1,026
-3,42	4,23
16,63	12,179
-3,91	3,738
1,892	5,886
0,813	3,049
-1,003	3,636
1,837	3,364
-1,908	2,125
-1,3	0,987
-2,899	0,608
17,757	9,281
-6,855	1,687
4,907	0,923
-4,301	4,046
-1,615	0,517
7,476	4,155
-0,175	2,789
0,517	1,79

-4,048	1,212
-0,082	2,464
26,723	11,955
5,865	0,358
-6,993	1,706
-3,374	1,096
-3,01	12,228
0,186	1,809
-0,965	2,945
-2,932	2,69
0,033	1,556
-1,541	1,862
-2,669	6,101
3,291	0,441
10,455	31,494
0,097	5,297
8,005	0,367
